# Typhoid Fever

**Published:** 1889-01

**Authors:** J. P. Stevens

**Affiliations:** Georgia


### Southern Medical Record.

A MONTHLY JOURNAL OF PRACTICAL MEDICINE.

All communications must be addressed to R. C. Word, Managing Editor.

" *Quicquid Præcipies Esto Brevis.*"

VOL. XIX ATLANTA, GA., JANUARY, 1889. NO. 1

### ORIGINAL AND SELECTED ARTICLES.

### TYPHOID FEVER.

By J. P. Stevens, m. d., of Georgia.

Read before Macon Medical Society, June 5th, 1888.

The study of Typhoid fever in its varied aspects as concerns its etiology, pathology, and treatment would require a volume for its proper elucidation. It would be impracticable for me in a short essay to present more than a cursory glance at the more important aspects of its literature, which have reference to practical results. The co-entity of typhus and typhoid fever has been advocated by many intelligent minds, but a careful annotation of the conditions necessary to the induction of either type of these fevers, clearly proves their distinctive individuality.

Typhus is evidently a purely contagious disease like any of the exanthemata. Typhoid fever is non contagious; not communicable by direct contact. Says Leibermeister, " whoever comes near or touches a case of typhus is in danger of contagion. For this reason a number of the physicians and attendants who take care of such invalids, are themselves, attacted by the disease. In Ireland in the year 1847, no less than 500 medical men, one-fifth of the entire number, suffered from typhus, and 127 died. In the Crimean war at the height of the epidemic, among the French, out of 840 attendants in twelve hospitals, 603 were taken sick in 57 days. More than 80 surgeons died in the course of the campaign. Complete isolation was the only protection.

Typhoid fever is non contagious: That is, the physicians and nurses are no more liable to attack than those who have never seen the disease. According to Murchison. "In 141-2 years in the London fever hospital 2.506 patients were treated and only 8 cases originated in the hospital." Leibermeister says: "Up to the year 1865 I have never seen in the hospitals which I visited (Grisswald, Berlin, Tubingen) a single hospital physician or nurse attacked with typhoid fever, although such cases are placed in the general wards. Other observers have had the same experience."

ETIOLOGY.—Typhoid fever is eminently infectious. The question arrises how does it originate?

From the putrefactive decomposition of organic matter. The presence of nitrogen is necessary to the generation of the specific poison through animal and vegetable fermentation. The same causal relation exists with reference to so-called malarial fever: to dyptheria, dysenterry, yellow fever etc. But typhoid fever is the product of a peculiar bacterium, the bacillus of Oberth, which produces the ptomaine typhotoxme, a toxic putrefactive chemical compound. The results of experiments upon inferior animals by Seitz, Vaughn and Novy, Bricger, Frankel and Simmonds, and others, clearly confirm the discovery in 1880, of Oberth, of the peculiar bacillus, which he claims to be the cause of typhoid fever.

One of the most fruitful sources of this poison is impure well water and milk, which has been exposed to the contagion, or subject to agitation while warm for several hours before use; particles of dust floating in the air and containing minute solid substances in suspension coming in contact with the pharynx alimentary canal, and by inhalation into the lungs; also by clothing and bed linen.

One of the most virile sources of infection is from the excrements of the patient, which, when recently voided, may be handled with comparative impurity, but in a few hours putrifactive fermentation begins, and the peculiar ptomaine is generated that becomes so conspicuously poisonous. Some years ago a destructive epidemic of typhoid fever prevailed at Eastman in this State, among the boarders of the large hotels occupied chiefly by Northern visitors. Many were stricken down and others were taken sick on the return to their homes. There was a search made for the cause of the fever and it was found that a break in the pipe that furnished water to tne building communicated with the affluvia that emanated from the water closets." "Epidemic in Solothurn 1865." In a locality suplied by a certain aqueduct, during the period between August 15th, and September 15th, a number of persons were attacked with typhoid fever; 82 of these cases are described with their names. Almost all the houses supplied with water from this acqueduct contained cases of typhoid, while other houses near and between these, but with a different water supply, escaped entirely. In the barracks which were supplied from the acqueduct, numerous cadets and instructors collected from different cantons were attacked with the disease, whioh commenced fourteen days after their moving into the barracks. In 11 days, 32 were attacked, other schools being then given up, after its dismisal ten more were attacked. Out of 100 persons, 42 were attacked with severe typhoid and 8 died. At the same time the disease appeared among other dwellers in the barracks which were supplied from the aqueduct, although before that time there had never been a case of typhoid there. It was found, on examination, that a brook which passed through the court of the lunatic asylum Rosigg, and received its sewage, ran into the aqueduct. In the asylum there was a nurse who had recently came from a typhoid locality. This woman was taken sick with typhoid fever about the middle of July and died August 8th. The clothes of this patient were washed in the washhouse of the asylum, by order of the director, and many of the soiled clothes were even soaked in the brook itself. After the middle of August, the epidemic appeared throughout the entire locality supplied by the aqueduct."

We have the following statement, in relation to an infected well, in Basle. In a collection of houses situated at some distance from the city, of which the inhabitants numbered about 150, mostly girls of thirteen to seventeen years old, there were no cause for typhoid during the severe epidemic in Basle in 1865 and 1866. In the year 1867, when the epidemic had subsided iu the city, a single case appeared in January: a second case in February, and in May a large number, so that within twenty days thirty-six persons were attacked with typhoid fever, and many others with febrile and afebrile catarrh. It was shown that the well from which the drinking water was drawn was fed from a canal into which emptied the privy. Eighteen days after this was forbiden there were no more new cases."

SEQULÆ.

Convalescence from typhoid fever like the prodroma is usually slow. Perversions in diet and too violent exercise are the most prolific causes of relapse, not unfrequently attended with intestinal perforations, and disastrous results. Sciatica followed by partial paralysis is sometimes a sequel of this disease. In my experience I remember that two cases of convalescence, where ultimate recovery was almost assured, suddenly terminated fatally through imprudence. One from entire change of clothing and sitting up in a chair, contrary to explicit instructions, and the other from indulging fondly in eating parched peanuts and drinking mean whiskey. Acute Brights disease is sometimes a sequel to this disease, but according to the best authority, it rarely ever passes into the chronic stage, and is almost uniformly curable. Acute catarrh of the bladder, orchitis epididymitis, metronorhagia, feruncles, glandular abscesses, various exanthematous affections, and bed sores, very often hinder the progress to complete recovery.

*Diaguosis.* An absolutely correct diaguosis of thisdisease is not always made. An eminent author says: "There is not a single symptom belonging to typhoid fever which can be characterized as pathognomonic. **It is hardly necessary to hear that it will afford but little assistence in making the diagnosis of typhoid fever, and in distinguishing it from other diseases, to lay down a series of dogmatic rules, which the unskilled physician will not know how to use rightly, and the skilled will not feel the need of." I believe that in general, physicians are too exclusive in confining their diagnosis to the uniform presence of the existence of iliae tenderness, pointing to local inflammatlon of the entire glands of Peyer and Brunner. In all cases of much intensity, prodromic stage is usually slow, lasting from a week to ten days before the patient is compelled to take his bed, during which a feeling of general malaise, anorexia and indisposition to physical cnd mental exercise, are the most prominent symptoms, Tenderness in the iliac region, enlargement of the spleen, roseola-slight furring of the tongue, stupor, and constipation of the bowels, are among the usual symptoms in the inception of the disease. As the stage progresses corresponding changes occur in the increase of intestinal irritation, a gradual increase of temperature, rapid metabol. ism of tissue, and change in the appearance of the tongue. The duration of the disease varies with the intensity of the attack and lasting from fourteen to forty or fifty days.

PATHOLOGY.

The lesions which occur are, first, those which result primarily from the specific poison; changes that occur in Peyer's glands and agminated patches and the lymphatics of the intestmal track; and secondly such lesions as are the general disease, i. e. degeneration of parenchymatous tissues in the heart, liver, kidneys, pancreas, and the voluntary muscles. The intensity of these lesions is dependent up[illegible] the intensily and duration of the general disease and especially t[illegible] of the fever. The course of the lesions may be divided into f[illegible] periods, of one week, each corresponding with the fractional parts [illegible] the usually recognized duration of a typhoid case of fe[illegible] " In the first week the mucous membrane of the intestine, princi[illegible]ly that surrounding Peyer's patches in the lower part of the ili[illegible] becomes hyperæmic and swollen. Gradually the *medullary infiltrat*[illegible] is developed in a certain number of Peyer's patches and solit[illegible] follicles ; not in all at once, but on the first day a few patches [illegible] follicles are swollen, and on the successive days more and more. [illegible] a rule, the infiltration begins in the neighborhood of the ilio-cæ[illegible] valve and extends to a distance from this on the following d[illegible] By the end of the first week all the patches are infiltrated which [illegible] likely to undergo that change.

In the second week, the hyperæmia of the mucous membrane [illegible]creases, the infiltration of Peyers patches continues to increase gra[illegible]ally, some of the swollen patches become partially or entirely *necrotic.* The *necrotic process*, with the formation of *sloughs* stained yellow by the bile, is nearly completed by the end of the second week. In the milder cases the necrotic process is trifling in extent, or even entirely absent; but at the end of the second week, or in abortive cases earlier, a retrograde process sets in. In the third week the sloughs fall off, leaving losses of substances of variable extent, useually extending down to the muscular coat. By the end of the third week the cleaning off of the ulcers is principally accomplished. In the 4th week the ulcers usually cicatrize, but this process may not be completed until some time later (Leibermeister). In the mesenteric glands we observe analogous changes. The rapidity and intensity of the parenchymatous tissue degeneration are dependent very much on the intensity of the fever. We observe also different grades of degeneration, the granular, the fatty and the waxy. The gravity of the symptoms is commensurate with these different grades of degeneration. The marked disturbance of the functions of the central nervous system and of the brain exhibited in great physical prostration, lethargy, tendency to chorea and often wild delirium, indicate the virulence of the poison that penetrates every fibre and tissue of the body. The intensity of these disturbances, at least in some cases, progress *pari passu* with the different weekly gradations observed in the progress of the disease.

PROPHYLACTIC TREATMENT.—Much may be done as accessory to the therapeutical use of remedies in the treatment of this disease. Even bearing in mind its microbic origin, such measures should be assiduously employed as will keep the atmosphere of the room in as healthy a condition as possible. A scrupulous regard to free and abundant ventilation is of the first importance. The air of the sick room laden with the debris of broken down tissues and in the bountiful exhalation of carbonic acid and watery vapor, we can readily see how the function of respiration and aeration of the superheated blood may greatly impair the functional activity of the vital organs and add to the distress of the patient. Some suitable disinfectant such as permanganate of potash, sulph. of iron, hypochlorite of soda in solution should always be kept in the sick chamber. The alvine dejections should be treated with a snlution of hypochlorite of soda, one drachm to the pint of water, after every dejection and burried in a trench 18 inches or two feet deep. All articles of clothing or bed linen which might have been soiled by the excreta should be boiled for an hour in order to the destruction of the infection. If not assured of the purity of the water for drinking purposes, it should be boiled before use. If practicable the patient should be removed from one room to another every twenty-four hours. When this is not practicable a change of beds once a day in the same room would be greatly to his advantage. The exclusion of visitors from the room where the patient is annoyed by them, and confining him to the care of a single intelligent nurse is highly desirable.

SPECIFIC TREATMENT.—By specific treatment is meant all those medicaments as well as external measures applied to tha patient for counteracting the septic poison and its distressing results. As we have no remedy which can be relied uyon in acting chemically upon the blood for the direct neutralization of the poison, we are left to treat the disease in accordance with the general symptoms and our knowledge of its pathology. We should even keep in mind that a persistently high temperature is indicative of a very grave type of fever, for it rapidly induces degeneration and waste of tissue, with general anemia and weakness of heart action, and a tendency to paralysis of the heart. A persistently high temperature for a number of days, say where the temperature keeps above 103, is almost surely accompanied by fatty degeneration of the muscular and glandular tissues with consequent dipression of the vital powers. The treatment should be commenced by clearing out the alimentary tract of all offensive debris caused by impaired glandular secretion of the liver as well as of the intestinal glands.

For this purpose there is nothing better than calomel in small and repeated doses until the intestinal canal is completely cleared of all offesive accumulations. As is well known, calomel is highly antiseptic, and its action is mild and unirritating. For the reduction of temperature, frequent sponging of the body, say once every half hour or hour, or where this is not sufficient, the general cold bath commencing with the temperature about 75 deg., and gradually lowering it, according to the tolerance of the patient for this mode of reduction; for some temperaments are much more susceptible to the action of cold than others. The patient may be kept in the cold bath from ten minutes to half an hour at a time. He should then be taken out, wiped dry, and if he should feel chilled, a glass of wine or whiskey may be administered. "In the hospital at Keil, during the years from 1850 to 1861 under varying and not very energetic treatment, there were 51 deaths among 330 typhoid fever patients, that is 15 4-10 per cent. During the years from 1863 to 1866, under systematic cold water treatment, there were five deaths among 160 such patients, thet 3 1-10 percent, and in still later times the results seem to be even more favorable than before." Antipyrin is one of the most potent agents in our possession for the reduction of temperature. As regards the dose one grain for every year of the age of the patient up to maturity is a very fair rule whereby to govern the size of the dose. We cannot say that this remedy has any specific effect in its curative agency, but the profuse perspiration which is induced especially relieves the capillaries of that degree of tension which is so exhaustive of vital energy, and thus aids the vital power in efforts at recuperation. It appears to me that I have seen its use followed by a turn of the disease towards convalescence. One of the most potent remedies in the treatment of typhoid fever is the tinct. of viratrum viridi. It lowers the temperature, slows the pulse and economizes force. It is is estimated by Hales that the force expended by the heart, at each beat, in propelling the blood into the aorta is equivalent to 4 1-2 lbs. Av. and with a pulse of 120 beats for minute and a reduction of it to 80 beats, we have a saving of force equivalent to 180 lbs. per minute. The energy therefore that is expended in this mechanical work of the heart, in obedience to the law of the correlation and indestructibility of force, passes into chemical force employed in the disintegration and waste of tissue, and thus the ver. viride acts indirectly as a tonic by retarding this waste, and saving so much of tissue to the economy. This conclusion is confirmed by a knowledge of the fact that the fullness and softness of the pulse, is proportional to the decrease in the number of heart beats.

In the anaemic stage where vital energy is at its lowest and there is danger of heart failure, the veratrum will not produce its characteristic effects, and hence is not indicated. It is best to commence with a small dose, three drops with twelvth or sixteenth of morphine and repeat every hour until the desired reduction is accomplished. This will prevent the distressing nausea and vomiting that sometimes follows large doses at more protracted intervals. The effects may be prolonged by continuing the remedy at longer intervals. I have employed veratrum in this and many other diseases for 30 years, and am yet to find the first case where it has done any harm, and uniformly its influence has been salutary. Where nausea and vomiting have been induced by excessive doses, and corresponding temporary depression, morphine and whiskey will soon cause a healthy reaction. The slowing of the pulse and consequent economy of force may be kept up for many days with no discomfort to the patient, but with positive quiescence of nervous perturbation. I might adduce many instances in confirmation of this truth. Sometimes we have prejudices against the use of certain remedies, like blood letting in acute inflammatory disorders, which has almost been consigned to the tomb of oblivion, superimposed by prejudice as its senseless epitaph. Among specific remedies,those which promise a direct influence in neutralizing or counteracting the effects of the microbic poison, is a solution of iodine and iodide of potassium; one part of iodine, two parts of iod. potassium and ten parts of water, giving three or four drops in water every two hours. I have employed this remedy and cannot vouch for its specific action, but can testify that no fatal case has followed its use. Whether *post hoc ergo propter hoc* is a legitimate conclusion, I am not prepared to say. As regards the use of quinine nothing can be said in its favor, except as an antipyretic in large doses, 20 to 30 grs., and antipyrine and antifebrine as well as cold effusion and cold immersion, are much more reliable as antipyretics, for they much less disturb the stomach and the sympathetic system of nerves. In the incipiency of this disease I cannot say that I have ever observed any beneficial results from quinine unless it be in combination with the salicylate Sod. In one case it appeared to be abortive, but whether the speedy termination of the disease was attributable to its legitimate termination in accordance with its type and grade, or to its action as a prophylactic I am unable to say. Too much stress cannot be placed upon proper hygienic measures and proper nutrition of the patient. We must even bear in mind that the tendency of the disease is to atrophy of the tissues, and consequent weakness of the vital energies and ultimately to death. In the solution of proper dietary appliances we must remember that there is a vast expenditure of vital force in the rapid disintegration of tissue, and that the gastric glands which are concerned in the digestion of albuminous substances are comparatively in abeyance. The final digestion of introgenous substances in the intestinal canal depends upon the conversion in the stomach of the albumen into albuminose in order to prepare it for pancreatic digestion and final assimilation to the organic tissues. The natural inference substantiated by experience is that animal food of all kinds is injurious. It is invariably followed by increase of temperature, nervous irritability and general disquietude, when consumed in its most digestible form, partially cooked or raw meat. The hydro. carbon would therefore come in the front rank of nutritive agents; those agents that are concerned in the conservation of animal heat, and in support of the respiratory functions. Recognizing the comparative torpidity of the gastric glands, it will readily occur to us that partially peptonized food, such as malted milk where the starch has been transformed by fermentation into glucose before use, Liebig's beef extract, lactopeptine, peptonized milk, butter milk, decoction of corn meal with lime water, and the support of flagging nervous energy by the heavier wines and alcoholic preparations, comprise the most desirable dietetic adjuvants to the conservative processes of the vis medicatrix naturæ. In summing up the general indications for treatment we will bear in mind that the disease is asthenic in its type, and its tendency is to death. A wise therapia therefore, would indicate not the expectant plan, leaving all to nature, but that plan which would prompt us to hold up her hands, study her methods, be guided by her counsels and support her by any available appliance in our reach. It is estimated by statistical data "that under the expectant treatment the mortality was 27 per cent, and under systematic antipyretic treatment the mortality was 16 per cent. Let us therefore heed the admonitions of intelligent experience, and strive after the attainment of comparative, if not absolute truth. Truth is seated high upon her royal throne, swaying an imperial sceptre, bringing into subserviency her exhaustless resources in the domain of philosophical and scientific research, and he who upon bended knees basely picks up the crumbs of ill-defined, blind and rejected hypotheses that fall from her lap, wanders about in doubt and obscurity, unconscious of the brilliant light that points to certitude, and applied knowledge, which is wisdom.

*Corns.*.—Shoemaker uses a salve made of equal parts of salicylic and lanoline.

